# Demyelinating Changes Alike to Multiple Sclerosis: A Case Report of Rare Manifestations of COVID-19

**DOI:** 10.1155/2020/6682251

**Published:** 2020-12-28

**Authors:** Fatemeh Yavari, Sara Raji, Fatemeh Moradi, Morteza Saeidi

**Affiliations:** ^1^Mashhad University of Medical Sciences, Mashhad, Iran; ^2^Student Research Committee, Faculty of Medicine, Mashhad University of Medical Sciences, Mashhad, Iran; ^3^Department of Neurology, Ghaem Hospital, Faculty of Medicine, Mashhad University of Medical Sciences, Mashhad, Iran

## Abstract

COVID-19, as a global concern and pivotal issue in the healthcare system, could have various presentations, leading to difficulty in diagnosis and management. Neuroinvasive potency, as claimed by preliminary studies, is a considerable pathogenesis. Serious neurological disorders like multiple sclerosis (MS) were out of the blue to be the first demonstration of COVID-19. This report highlights the representation of MS in a young woman, which resulted in a COVID-19 diagnosis.

## 1. Introduction

In the shadow of a global pandemic of COVID-19, researchers and clinicians recently identified neurological manifestations in some patients peculiarly who suffered from severe diseases and had older ages [1–5]. Regarding reports of cases with demyelinating lesions and encephalitis in the context of COVID-19, this disease might mimic known conditions such as multiple sclerosis [6]. MS is an unexpected presentation of COVID-19 which had not been reported as the first demonstration of this infection. This study aims to represent a young woman with an established diagnosis of COVID-19 appearing with multiple sclerosis manifestations.

## 2. Case Presentation

A former healthy 24-year-old woman with no family history of neurological problems presented to the neurology clinic complaining of sore throat, low-grade fever, and myalgia since last month. The patient displayed no respiratory symptoms, such as cough and dyspnea. Next, blurred vision and diplopia were added to her symptoms, and the severity of diplopia gradually increased within one day. Continuously, fever, sore throat, and myalgia persisted. Afterward, she developed anosmia, drooping at the corner of the left lip, paresthesia of the fingertips in both upper extremities, left eyelid drooping, and left eyebrow sagging of left facial nerve involvement. Despite the delay due to the phobia of confronting incurable diseases, she finally got the neurologist appointment.

On physical examination, the blood pressure was 110/75 mmHg, the heart rate 70 beats per minute, the temperature 37.7°C, the respiratory rate 12 breaths per minute, and the oxygen saturation 94%. The Mini-Mental State Examination (MMSE) was within the normal range. Her pupils reacted equally to the light. There was no evidence of poppy edema on fundoscopy, neither increased intracranial pressure nor meningeal irritation signs. Except for bilateral anosmia and left-sided nerve facial paresis, other cranial nerve examinations were unremarkable. The muscular strength was 5/5 in all four limbs; also, muscle tone was normal. The deep tendon reflexes and flexor plantar response were normal. We did not detect any sensory levels in the patient. Besides, there was no evidence of urinary or fecal incontinence.

Magnetic resonance imaging (MRI) was notable for multiple plaques in different brain areas with hypersignal intensity in T2 and fluid-attenuated inversion recovery (FLAIR) sequences. Subsequently, laboratory markers tests such as ANA profile (vasculitis test), tuberculin test (PPD), Coombs Wright & 2ME tests, and Venereal Disease Research Laboratory (VDRL) test were performed. Moreover, routine viral markers such as the varicella-zoster virus antibody (VZV-Ab), hepatitis B virus antigen and antibody (HBV-Ag and HBV-Ab), hepatitis C virus antibody (HCV-Ab), and human immunodeficiency virus (HIV) type 1 and type 2 antibodies, as well as human T-cell lymphotropic virus type 1 (HTLV-1) antibody, were checked which had no significant results. Also, erythrocyte sedimentation rate (ESR) was 10 mm/h.

**Figure 1:**
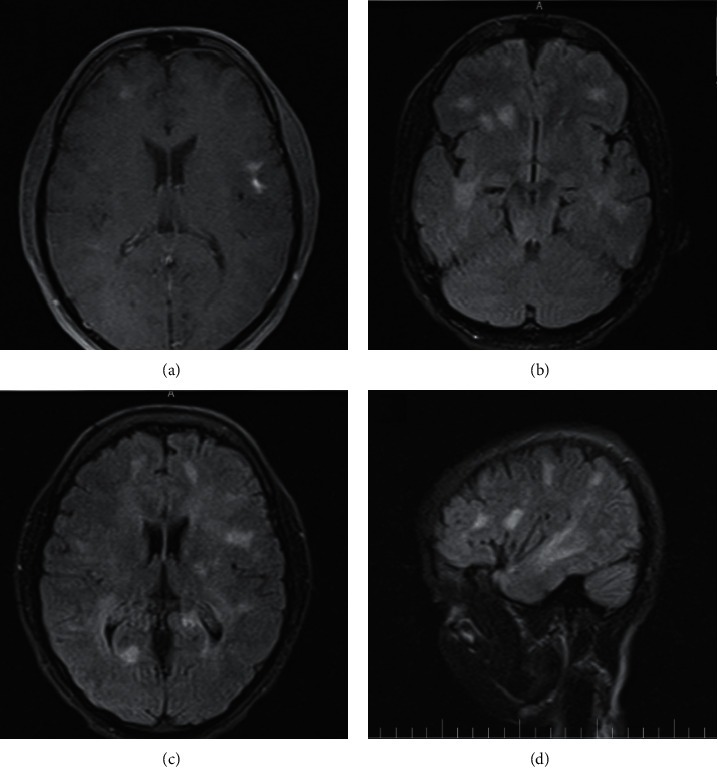
(a) Head-MRI T1 sequence with gadolinium demonstrates various hypersignal lesions in favor of active demyelinating plaques in left temporal and right frontal areas of the brain. (b, c) Axial and (d) sagittal FLAIR views show plaques with larger sizes in deeper areas of the brain.

Regarding multiple hypersignal plaques reported in MRI with the suspicion of demyelinating processes such as multiple sclerosis (MS), full-field pattern reversal visual evoked potential (PRVEP) test and brain and cervical MRI with and without gadolinium had been undertaken. The VEP did not indicate any abnormal visual pathways. The MRI again demonstrated multiple plaques with hypersignal intensity on T2-weighted and FLAIR images plus active plaques on T1 with contrast (Figure 1). According to the patient's clinical manifestations and the existence of reported plaques, the diagnosis of multiple sclerosis had been clinically suspected. Furthermore, the patient fits McDonald criteria, one clinical attack plus more than two lesions with objective clinical evidence and dissemination in time evident on MRI (appearance of active plaques in T1 with Gd and plaques in T2); hence, pulse therapy with 1 g methylprednisolone with dextrose 5% in water daily divided into two doses for four days was started. After initiating the treatment, fingertips paresthesia and facial nerve paresis improved, without any resolution of intermittent fever and anosmia. Concerning lack of improvement in myalgia, anosmia, and intermittent fever plus occasional dyspnea, as well as being in the era of COVID-19 pandemic, we retrograde re-evaluated MRI and hesitated to COVID-19. Following this, we discovered the atypical pattern of the plaques in MRI, which were larger than the routine plaques found in MS patients. In addition to common areas such as periventricular and juxtacortical, the plaques were established deeper and farther from periventricular and juxtacortical areas. Hence, a nasopharyngeal sample of the patient was taken for real-time polymerase chain reaction (RT-PCR) assay, which tested positive for SARS-CoV-2. This result prompted the lung high-resolution computed tomography (HRCT), which was notable for bilateral diffuse ground-glass opacities for SARS-CoV-2 pneumonia.

Due to the possible diagnosis of COVID-19 neurological complications, the treatment was started with azithromycin 500 mg daily (first day BID) and naproxen 500 mg BID for one week. About maintenance therapy, we decided to order a drug covering both MS and COVID-19 symptoms; thus, subcutaneous interferon-beta-1a (Resigen, continued for three months) three times a week was chosen consensually.

Furthermore, escitalopram 10 mg daily and clonazepam 0.5 mg daily were added to control the patient's anxiety. In the follow-up, the patient somewhat recovered except for an occasional nonproductive cough. After three months of therapy, we will evaluate the patient's status and take an MRI to decide appropriately for further long-term treatment.

## 3. Discussion

The widespread COVID-19 crisis, with its various manifestations, causes a striking burden in the health system since some patients do not represent typical symptoms and signs like fever or ground-glass opacity in CT scans [7]. Although SARS-CoV-2 mostly affects the respiratory tract, extrarespiratory involvements, including gastrointestinal, cardiac, renal, hepatic, hematological, dermatological, and neurological, can occur. Recently, plausible documents suggested the neurotropic feature of COVID-19 and its effects on the nervous system as a principal organ [3, 8, 9]. It is assumed that human coronaviruses' neurovirulence might contribute to short- and long-term neurological diseases such as encephalomyelitis and multiple sclerosis. As they longer persist in the CNS, the more long-term sequelae could develop [10]. There is mounting evidence that focused on similarities between coronaviruses and their neurotropism features, which could be applicable for SARS-CoV-2 [4]. Mindfully, to elucidate how COVID-19 acts as a neuropathogen, some authors blamed various mechanisms of neurotropism of COVID-19. The virus in blood circulation as a direct pathway might enhance the permeability of the blood-brain barrier via cytokines produced from macrophages, which promote it to penetrate the brain; however, the evidence is inadequate to prove this hypothesis.

Moreover, COVID-19 could directly invade the CNS through the olfactory tract and cause an altering sense of smell and demyelinating destruction. Indirectly, hypoxia can occur due to inflammatory exudate by virus proliferation in lung tissue cells and alveolar gas exchange, which puts patients at risk of cerebrovascular accidents. Also, COVID-19 elevates blood pressure by binding to angiotensin-converting enzyme 2 (ACE2) receptors that predispose the patients to develop ischemic stroke and cerebral hemorrhage and coagulopathic side effects. Lastly, cytokine storm syndrome, as a known consequence of COVID-19, might account for a hyperimmune response, which results in chronic inflammation and CNS damage [5, 9, 11, 12].

The most common brain lesions reported in COVID-19 patients were white matter hyperintensities on MRI and hypodensities on CT scan, specifically adjacent to ventricles. Nevertheless, a shortage of brain images on all patients might alter the results. Also, the deeper brain structures, cerebellum, and midline involvement were rarely reported [13].

Neurologic alterations in COVID-19 patients described in the literature included viral encephalitis, toxic encephalopathy, acute cerebrovascular disease, multiple sclerosis, Guillain–Barre syndrome, acute disseminated encephalomyelitis (ADEM), and peripheral nerve disease [5, 9, 12]. To clarify coronavirus's role, which may affect the autoimmune process in multiple sclerosis, some experimental studies had been done [5].

Duffy et al. believed Toll-like receptors might take part in MS pathophysiology via contributing to host defense and identifying detrimental microorganisms. Likewise, these receptors were represented to recognize those viral pathogens to modify patients' immune response suffering from MS. For instance, some authors reported a notable prevalence of defined strains of coronavirus such as 229E and OC43 in brain samples of MS patients. Moreover, in some murine experimental research, inoculated mice with coronavirus developed acute encephalomyelitis through a chronic demyelinating process; also, macrophages and oligodendrocytes invasion by autoimmune triggering against myelin protein was detected. Of note, a correlation between SARS-CoV-2 and MS has not been determined [5, 14]. The demyelinating plaques in our patient's MRI, with suspicion of MS, have been represented atypically. They had two dominant features that distinguished them from routine plaques defined by McDonald criteria previously. First, the plaques were overall larger than typical plaques. Second, the places where the plaques have appeared were deeper and farther from juxtacortical, infratentorial, and periventricular areas than the typical plaques. Our study's potential limitation was lacking CSF analysis due to the patient's discontent to perform a lumbar puncture. Hence, concerning the patient's autonomy and ethical issues, we could not interpret CSF analysis.

Compatible findings with COVID-19 in CT scan and suggestive clinical manifestations with positive PCR strengthened our study to confirm COVID-19 diagnosis in the patient. COVID-19 pandemic leads scientists to consider drug repurposing of such antiviral options with potential effects to defeat SARS-CoV-2 quickly. Interferon (IFN) *β*-1a, which is known as a beneficial therapy in MS, could have a significant therapeutic impact in COVID-19 patients [15]. According to Clementi et al.'s study, IFN-*β*-1a could effectively have an inhibitor role in virus-contaminated cells [15]. Furthermore, it was recommended to use IFN-*β*-1a therapy to preserve functional B-cell-mediated humoral immunity in MS patients in the COVID-19 era [16]. As we utilize IFN-*β*-1a with a brand name of Resigen in the first-line therapy of MS patients, we initiated it in our patient, which resulted in a beneficial outcome [16]. Despite all these findings, further and strong surveys are necessary to investigate the correlation between MS and SARS-CoV-2 for possible pathophysiology and appropriate management. Eventually, considering COVID-19 in patients with such neurological manifestations is pivotal to avoid detrimental outcomes and complications.

## 4. Conclusion

COVID-19, with its neurotropism feature, might appear in the first place of some neurological disorders like multiple sclerosis which require further evidence to establish causality.

## Data Availability

The supporting data for the findings in this study are available by contacting the corresponding author.
